# Exercise promotes cognition and hippocampal mitochondrial complex II expression in female rats

**DOI:** 10.1113/EP092533

**Published:** 2025-04-01

**Authors:** Zachary J. White, Keshari H. Sudasinghe, David C. Poole, Stephanie E. Hall

**Affiliations:** ^1^ Department of Anatomy and Physiology, College of Veterinary Medicine Kansas State University Manhattan Kansas USA; ^2^ Department of Kinesiology, College of Health and Human Sciences Kansas State University Manhattan Kansas USA

**Keywords:** complex II, coordination, exercise, hippocampus, memory, mitochondria

## Abstract

Evidence supports that exercise defends against age‐associated declines in brain health and protects against neurodegenerative disease. To help understand the molecular basis for the neuroprotection, we examined the impact of training on mitochondrial protein expression within the exercise–brain axis. Thirty‐two F344 rats (50% male/female) were assigned randomly to 10‐week treadmill training or sedentary groups. Grip strength, Morris water maze and rotarod were used to assess muscular strength, spatial learning and motor coordination, respectively. Jess automated western blotting was used to quantify mitochondrial complex expression in hippocampus and soleus skeletal muscle samples. Values are means and standard deviation. Exercised females had better spatial memory (9.16 ± 8.70 vs. 32.7 ± 22.7 s, *P* = 0.043) and motor coordination (69.0 ± 16.1 vs. 47.5 ± 15.6 s, *P* = 0.042) as well as increased soleus mass (0.043 ± 0.003 vs. 0.039 ± 0.002% body mass, *P* = 0.039), hippocampal mitochondrial complex II expression (1.96 ± 0.38 vs. 1.11 ± 0.33 a.u., *P* = 0.007), and soleus mitochondrial complex III expression (6.68 ± 1.40 vs. 4.65 ± 1.26 a.u., *P* = 0.025) in comparison to sedentary females. Cognitive performance and hippocampal metabolic enzyme expression were concordantly increased following the 10‐week exercise intervention in females but not males. These results provide novel support for the putative involvement of cerebral mitochondrial function in the beneficial relationship between exercise and brain health.

## 1 INTRODUCTION

Physical activity prevents and slows the age‐related decay of white and grey matter in the brain (Erickson et al., 2009, 2011). With natural ageing the human hippocampus undergoes a 1–2% decline in volume each year (Venturelli et al., 2011). While more research is needed, it is clear that exercise can not only prevent this decline, it can reverse it. Specifically, Erickson et al. (2011) found a 2% increase in hippocampus volume following a 12‐month exercise regimen in healthy older adults. Along with attenuating brain atrophy, exercise can effectively improve memory and cognition in older adults (Cheng et al., 2014; Erickson et al., 2009). Indeed, exercise provides an efficacious defence against age‐related cognitive decline and is suggested as a viable strategy to reduce AD prevalence (Huuha et al., 2022). Despite this work, we are far from understanding the mechanistic bases for the effects of exercise on brain function.

Significant work has been done to highlight exercise‐induced mitochondrial function improvements, specifically improving mitochondrial biogenesis and respiration, with recent discoveries uncovering the importance of the respiratory chain supercomplex formation. Greggio et al. (2017) found that exercise leads to supercomplex assembly and importantly that exercise differentially modulates skeletal muscle electron transport chain (ETC) complexes. This novel finding sheds light on the importance of evaluating ETC complexes individually, but very little is understood about how exercise might impact the ETC in other tissues, specifically the brain. While it is known that exercise can improve brain mitochondrial biogenesis and ETC coupling in the brain, this is the first investigation to quantify mitochondrial complexes in the brain following exercise (Gusdon et al., 2017; Steiner et al., 2011).

Herein, we report the effect of an exercise protocol administered in healthy male and female rats on memory, coordination, strength and mitochondrial complex expression in the hippocampus and skeletal muscle. The purpose of this investigation was to evaluate the exercise–brain axis in both males and females. We hypothesized that exercise‐induced cognitive benefits will occur concomitantly with hippocampal and skeletal muscle mitochondrial changes.

## 2 METHODS

### 2.1 Experimental animals

Thirty‐two Fisher 344 rats (16 males, 16 females) were acquired from Charles River (Wilmington, MA) and housed in the Comparative Medicine Group animal facility at Kansas State University accredited by AAALAC International (000667), OLAW (A3609‐01) and USDA (48‐R‐0001) assured. Animals were housed two to three per cage on a 12:12 h light:dark cycle and given free access to food and water. All procedures were in accordance with the *Guide for the Care and Use of Laboratory Animals* under the approval of the Animals Care and Use Committee at Kansas State University (protocol code 4466 and date of approval 9 October 2020).

### 2.2 Treadmill training

At 8 weeks old, animals were randomly assigned to sedentary cage activity or treadmill training exercise intervention. Following 1 week of acclimatization, a 10‐week (5 days per week) treadmill training protocol was completed during their dark cycle. Training intensity progressed to 36 m/min at 0% incline for 60 min per day by week 9 (Table 1). Similar protocols have been shown to elicit ∼75% of maximum oxygen consumption (Brooks & White, 1978) and induce both citrate synthase activity (Poole et al., 1989) and maximal oxidative capacity changes (Dudley et al., 1982) in soleus skeletal muscle. The training protocol was completed by 94% of the animals who entered; one female was removed from the study due to poor treadmill performance. Poor performance was defined at failure to complete at least 4 days of treadmill training in a given week.

**TABLE 1:** Ten‐week treadmill training protocol.

	Week									
	1	2	3	4	5	6	7	8	9	10
Speed (m/min)	12	20	22	24	26	30	33	33	36	36
Duration (min)	15	30	40	45	50	50	55	55	60	60

### 2.3 Behavioral testing

All behavioural testing was completed by a researcher blinded to the animals’ treadmill/sedentary assignment. Behavioural testing was completed prior to training during the last 2 days of the treadmill training protocol.

#### 2.3.1 Grip strength

Similar to human hand‐grip tests, rodent grip strength can be used as an indirect measure of total body strength. To assess grip strength, each animal completed three trials separated by 15‐min intervals during the animals’ dark cycle in the last week of the treadmill training intervention. In each trial, the animal was gently lowered over the top of the rectangular grid so that all four paws gripped the grid. Once the animal secured their attachment to the grid, the rat was pulled back steadily with its torso kept horizontal until the grip was released. Grip strength was measured as the maximal force (grams) the animal was able to withstand prior to releasing the grid and was recorded. The highest grip strength achieved during the three trials was calculated relative to body mass (grams force/grams body mass) and used for analysis.

#### 2.3.2 Rotarod

Motor coordination was assessed in all animals using a ramp rotarod protocol following grip strength testing. After sufficient acclimation with the rod, each trial was set up by placing one animal on the rod set at a constant rotation of 4 rpm. With the animal facing forward, acceleration was initiated and progressed from 4 to 40 rpm in 120 s. The trial began when acceleration started and ended when the animal fell from the rod. The trial length was automatically measured by the apparatus. The procedure was repeated for a total of three trials separated by 15‐min intervals. The highest latency time to fall for each animal was used for analysis.

#### 2.3.3 Morris water maze

Spatial learning and memory were assessed using the Morris water maze (MWM) on the day following grip strength and rotarod assessments, as described previously (Vorhees & Williams, 2006). Briefly, this MWM protocol consisted of an acclimatization period, learning trials and one test trial. During learning and test trials, the tank (180 cm diameter) was filled with water to a level that was 2‐3 cm above the platform, and non‐fat dry milk was used to make the water opaque. Under a red light and at a water temperature of 25°C, the animal was placed in the pool and allowed 60 s to find the hidden platform using the visual cues learned during the acclimatization period. A total of four learning trials were completed and timed. Learning trials were completed consecutively by each animal and began from each of the four quadrants. The animal was directed to the platform if it was not able to find the platform within 60 s on its own. Upon completion of the last learning trial, the animal was dried and returned to their cage. One hour following the final learning trial, a test trial was performed. In the test trial, the animal began in the quadrant opposite the platform. The latency time during the test trial was recorded and used for analysis. While latency time is viewed as the classical metric, it holds a strong correlation to video tracking data, time spent in target quadrant and platform crossings (Ménard & Quirion, 2012).

### 2.4 Tissue harvesting

Twenty‐four hours following their final treadmill training bout, animals were euthanized (isoflurane followed by decapitation) and tissues were collected. The brain was excised and weighed before the isolation of the frontal cortex and hippocampi. The hippocampi, once isolated, were lightly blotted and weighed. Soleus skeletal muscle was also excised and weighed. All tissues were first frozen in liquid nitrogen and then transferred to a −80°C freezer for later processing.

### 2.5 Protein quantification

Mitochondrial ETC complex subunits were quantified as an indicator of individual complex expression and mitochondrial health. Hippocampi and soleus tissue samples were lysed in radio‐immune precipitation assay (RIPA) buffer (Sigma‐Aldrich, St Louis, MO, USA) with protease inhibitor (Pierce Protease Inhibitor Mini Tablet, Thermo Fisher Scientific, Waltham, MA, USA) prepared according to the manufacturer's guidelines. Lysate protein concentrations were determined using the Qubit 4 Fluorometer with Protein Broad Range Assay (Thermo Fisher Scientific). Protein quantification was completed with Jess Simple Western (ProteinSimple, San Jose, CA, USA) automated western blot analysis with the following primary antibodies: mitochondrial complex I subunit NDUFS1 (60153, Cell Signaling Technology, Danvers, MA, USA), mitochondrial complex II subunit SDHA (11998, Cell Signaling, Danvers, MA, USA), mitochondrial complex III subunit UQCRFS1/RISP (95231, Cell Signaling Technology), and mitochondrial complex IV subunit MTCO1 (PA579700, Thermo Fisher Scientific). Lysate protein concentrations were equalized before protein quantification and each target protein was assessed in a single trial to ensure equivalent primary antibody exposure across hippocampus and soleus lysates, thus removing the need for a housekeeping protein. Protein expression was quantified with Compass for Simple Western software (San Jose, CA).

### 2.6 Statistical analysis

Ordinary two‐way ANOVA was performed to assess main effects of sex and exercise training. Šídák's multiple comparisons test with a single pooled variance was used to identify group differences. All datasets were normally distributed with the exception of body mass, brain mass, hippocampus mass, MWM latency time, hippocampus complex I and hippocampus complex IV. All analyses were performed using GraphPad Prism version 10.2.2 (GraphPad Software, Boston, MA, USA) using *P *< 0.05 as the level of significance.

## 3 RESULTS

### 3.1 Baseline characteristics

Prior to the 10‐week intervention, behavioural pre‐testing was completed and revealed no significant differences between groups (data not shown).

### 3.2 Exercise improved performance in tasks of spatial memory and coordination

**FIGURE 1:**
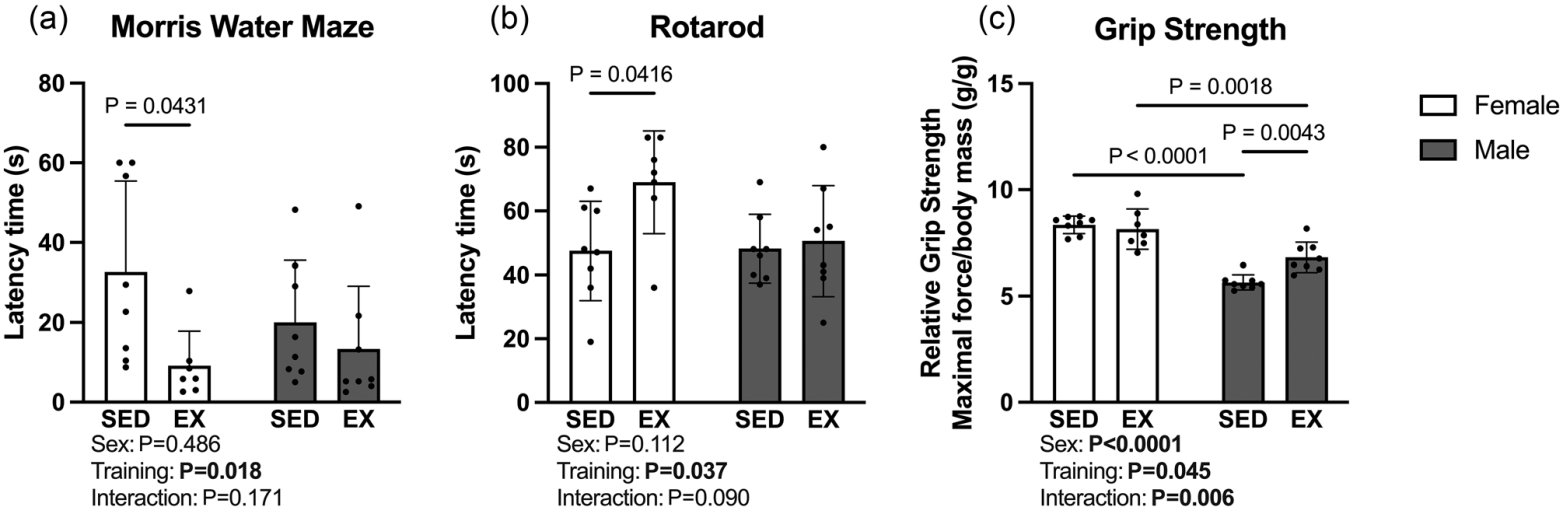
Results of behavioural tasks in males and females following the 10‐week treadmill training protocol versus sedentary. (a, b) Treadmill trained females displayed significantly better performance on tasks of spatial learning and memory (a) and coordination (b) compared to sedentary females while no differences were detected between male groups. (c) Grip strength relative to body mass was improved by exercise in males while an exercise‐induced group difference was not present in females. Values are presented as individual data with means ± SD (*n* = 7–8).

The MWM is a test of spatial memory with a lower latency time indicative of better memory performance. A significant main effect of exercise training was present (*F* (1, 27) = 6.36, *P* = 0.018, η^2^ = 0.178), with spatial memory performance being better in exercise trained animals. While both exercise groups had decreased latency time to the platform, only the females displayed significantly better memory than their sedentary counterparts (*P* = 0.043, Figure 1a). Similarly, on the ramp rotarod protocol, exercised animals displayed better motor coordination as a significant main effect of training presented (*F* (1, 27) = 4.81, *P* = 0.037, η^2^ = 0.130). Further, female exercisers displayed significantly better motor coordination than sedentary females (*P *< 0.042), while the males did not (Figure 1b). In the relative grip strength test, although main effects of both sex (*F* (1, 27) = 76.2, *P *< 0.0001, η^2^ = 0.647) and exercise training (*F* (1, 27) = 4.40, *P* = 0.045, *η* ^2^ = 0.037) were present, these main effects are difficult to interpret due to a significant interaction (*F* (1, 27) = 8.94, *P* = 0.006, η^2^ = 0.076). Sedentary (*P *< 0.0001) and exercised (*P* = 0.002) females displayed higher relative grip strength in comparison to male counterparts. In addition, relative grip strength was greater in exercise trained compared to sedentary males (*P* = 0.004, Figure 1c).

### 3.3 Exercise induces increases in skeletal muscle mass

**FIGURE 2:**
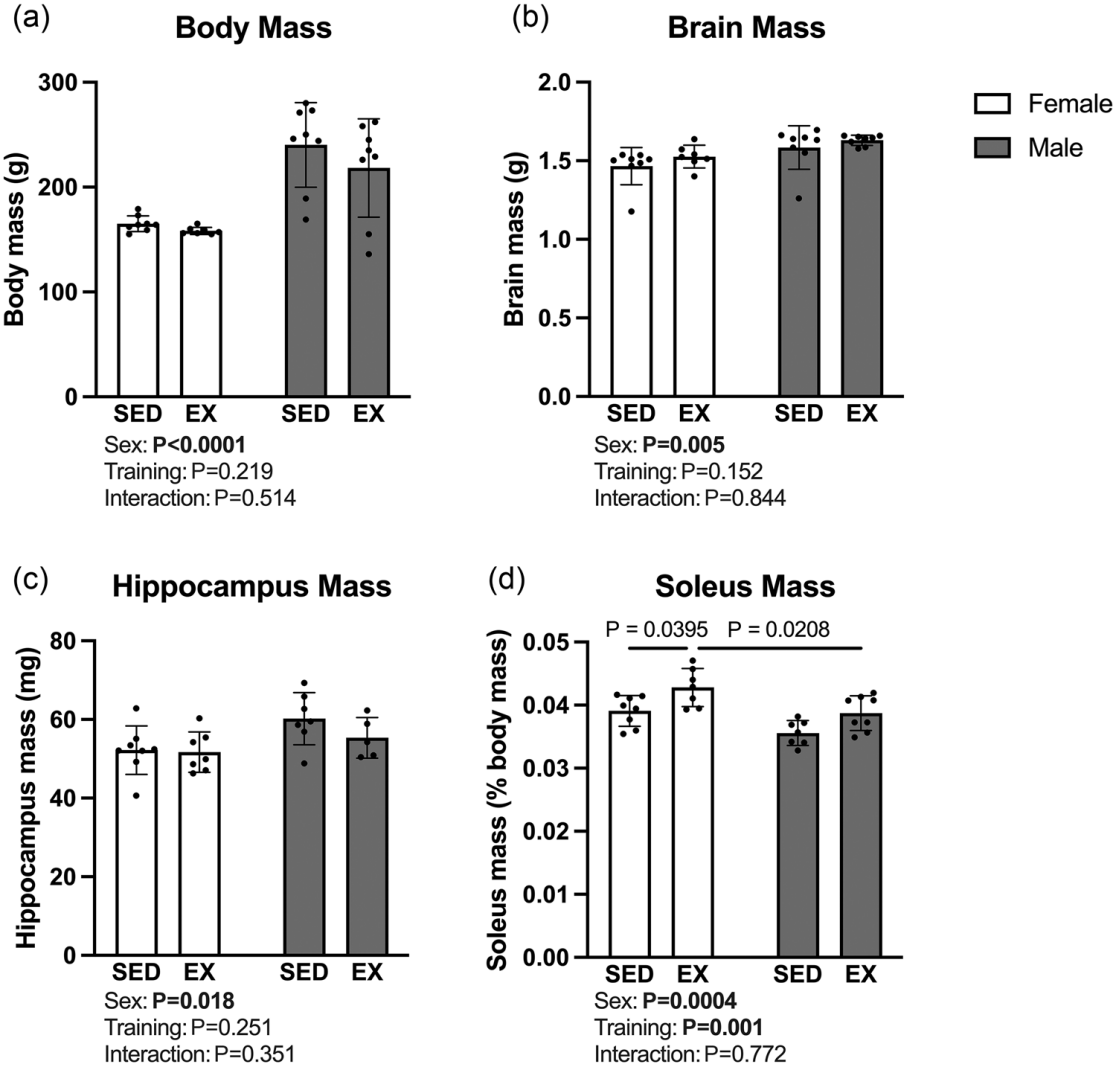
Between‐group comparisons of body mass and relative brain, hippocampus and soleus mass following a 10‐week treadmill training protocol in males and females. (a–c) Body mass, brain mass and hippocampus mass were not different between exercise and sedentary groups. (d) Increased soleus mass relative to body mass was seen in the female exercise group compared to both female sedentary and male exercise counterparts. Values are normalized as indicated and presented as individual data with means ± SD (*n* = 5–8).

Comparisons of body, brain, hippocampus and soleus mass are displayed in Figure 2. Importantly, body weight was not different between exercise and sedentary groups (Figure 2a). As expected, there was a significant main effect of sex in our analyses of body (*F* (1, 27) = 34.8, *P *< 0.0001, η^2^ = 0.546), brain (*F* (1, 27) = 9.49, *P *< 0.01, η^2^ = 0.243) and hippocampus (*F* (1, 23) = 6.48, *P* = 0.018, η^2^ = 0.199) mass (Figure 2a–c). The rat soleus is a hindlimb ankle extensor muscle composed predominantly of type I muscle fibres (Ariano et al., 1973). Accordingly, the soleus is sensitive to treadmill training‐induced adaptation, as previously demonstrated in similar training protocols (Dudley et al., 1982; Poole et al., 1989). Main effects of both sex (*F* (1, 22) = 16.1, *P* = 0.0004, η^2^ = 0.301) and training (*F* (1, 26) = 13.2, *P* = 0.001, η^2^ = 0.246) were present in our analysis of soleus mass as a percentage of body mass. Further, relative soleus mass was increased in female exercisers in comparison to both female sedentary (*P* = 0.039) and male exercise (*P *< 0.021) counterparts (Figure 2d).

### 3.4 Metabolic enzyme expression was upregulated in treadmill‐trained females

**FIGURE 3:**
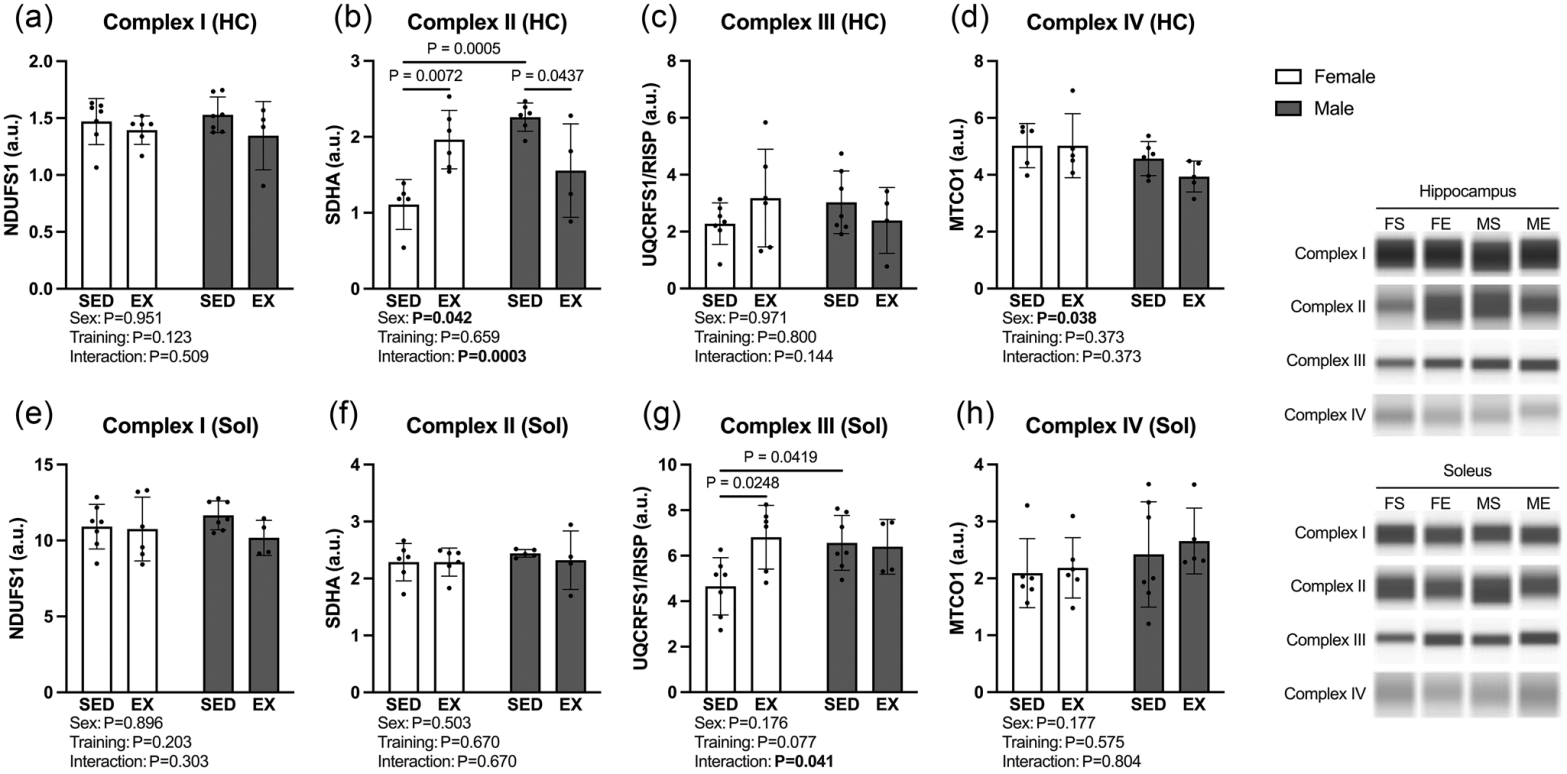
Metabolic enzyme expression analysis of complex I, complex II, complex III and complex IV in the hippocampus (a–d) and soleus (e–h) compared between groups. Increased hippocampal complex II expression was present in the female exercise group compared to sedentary females while male exercises significantly decreased complex II expression (b). Increased soleus complex III expression presented in exercise females and sedentary males in comparison to sedentary females. Values are presented as individual data with means ± SD (*n* = 4–7). a.u., arbitrary units; HC, hippocampus; MTCO1, mitochondrial complex IV subunit; NDUFS1, mitochondrial complex I subunit; SHDA, mitochondrial complex II subunit; Sol, soleus; UQCRFSI/RISP, mitochondrial complex III subunit.

Protein quantification of mitochondrial metabolic enzyme complex I, complex II, complex III and complex IV following treadmill training showed tissue‐ and sex‐specific differences between groups, as displayed in Figure 3. Our analysis of hippocampal complex II expression (Figure 3b) revealed a main effect of sex (*F* (1, 17) = 4.83, *P* = 0.042, η^2^ = 0.108), with expression being higher in males, and an interaction effect (*F* (1, 17) = 21.2, *P* = 0.0003, η^2^ = 0.476). Further, within the hippocampus of female exercisers, expression of mitochondrial complex II was increased compared to sedentary females (*P* = 0.007). Interestingly, in marked contrast, hippocampal expression of mitochondrial complex II was increased in sedentary males in comparison to male exercisers (*P* = 0.044) and sedentary females (*P* = 0.0005). Analysis of complex III expression in the soleus (Figure 3g) revealed a significant interaction effect (*F* (1, 20) = 4.80, *P* = 0.040, η^2^ = 0.149) with expression in sedentary females being significantly less than female exercisers (*P* = 0.025) and sedentary males (*P* = 0.042).

## 4 DISCUSSION

In the present investigation, female exercisers improved memory and coordination as a result of a 10‐week progressive treadmill training protocol while males did not. It has been noted previously that females tend to swim faster during the MWM; however, swim speed did not translate to improved (i.e., reduced) latency time as there were no noted differences in memory between male and female rodents (Zorzo et al., 2024). In a study utilizing voluntary running wheels, both sexes improved memory performance following 8 weeks of voluntary wheel running (Clark et al., 2008). However, recent meta‐analysis suggests males experience greater exercise‐induced improvements in non‐spatial memory tasks (such as recognition or conditional memory) as a result of wheel running and females improve hippocampus‐dependent learning and memory more than males as a result of treadmill exercise (Barha et al., 2017). Regarding motor coordination, females (both sedentary and trained) performed better on the rotarod compared to males (Clark et al., 2008).

During oxidative phosphorylation, the mitochondrial ETC diverts protons and electrons from NADH and FADH_2_ to reduce O_2_. These reactions occur gradually across four ETC complexes and drive the synthesis of ATP by complex V. Previous work has shown treadmill running to elicit proportionate increases in all mitochondrial proteins in rat skeletal muscle; however, in the current study, only hippocampal complex II was upregulated in female exercisers and, interestingly, was downregulated in male exercisers (Davies et al., 1981). This is important as the trained females displayed improved memory and coordination while no differences were found between trained and sedentary male groups. Among the ETC complexes, complex II, also called succinate dehydrogenase (SDH), has multiple distinguishing characteristics. Complex II is the sole ETC complex which oxidizes substrates in both the TCA cycle and oxidative phosphorylation. Within the TCA cycle, SDH oxidizes succinate to fumarate while reducing FAD to FADH_2_. Subsequently, complex II uses FADH_2_ as the electron donor for the reduction of ubiquinone to ubiquinol, the substrate of complex III. Due to this pivotal role, there is tight regulation of its biogenesis and function (Bezawork‐Geleta et al., 2017; Wang et al., 2023). Further, complexes I, III, IV and V have subunits encoded by nuclear and mitochondrial genomes, while complex II is comprised of four subunits encoded by nuclear DNA (Bandara et al., 2021; Bezawork‐Geleta et al., 2017; Wang et al., 2023). Complex II declines are associated with ageing and AD more so than any other mitochondrial complex (Adlimoghaddam et al., 2022).

The present investigation found sex differences in cognitive function and mitochondrial protein expression (complex II) following a 10‐week treadmill training protocol. Specifically, improved cognitive function (Figure 1a), coordination (Figure 1b) and metabolic protein upregulation (Figure 3b,g) were present in females but not in males. This may be due to multiple factors. Recent meta‐analysis suggests aerobic training may promote a larger increase in brain‐derived neurotrophic factor levels in female rodents compared to males (Barha et al., 2017). Additional work found female‐specific increases in cortical capillary length and total surface area following a 4‐month treadmill training intervention (Huang et al., 2013). Importantly, Aguiar et al. (2018) demonstrated unchanged submaximal ${\dot{V}}_{O_{2}}$ values across different phases of the oestrous cycle in female mice during moderate‐ to severe‐intensity treadmill running (Aguiar et al., 2018). Testosterone, in addition to the classical anabolic actions of increasing muscular strength and size (Bhasin et al., 1996), promotes aerobic endurance. Inhibition of androgen receptor signalling resulted in decreased time to exhaustion during a submaximal running endurance test in male rats (Georgieva et al., 2017). These factors, in addition to the previously discussed different influences of training modality (treadmill vs. voluntary running wheel) on cognitive domains between males and females, begin to explain the female‐specific influence of a 10‐week treadmill training protocol in the present work. Literature regarding sex influences on health processes has expanded vastly following the 2014 National Institutes of Health policy requiring preclinical research to consider sex as a biological variable. Further work is needed to understand the mechanistic basis of sex‐specificity within the beneficial influence of aerobic exercise on the brain.

### 4.1 Conclusions

This analysis demonstrates that exercise promotes cognitive performance and upregulation of mitochondrial protein expression (complex II) in the hippocampus of female rats. These results provide evidence for the involvement of hippocampal mitochondrial function in the beneficial relationship between exercise and brain health. Follow‐up experimentation is needed utilizing respirometry techniques to quantify the exercise‐induced improvements in cerebral mitochondrial function in relation to cognitive changes.

## AUTHOR CONTRIBUTIONS

Conceptualization, Zachary J. White, Keshari H. Sudasinghe, David C. Poole, Stephanie E. Hall; methodology, Zachary J. White, Keshari H. Sudasinghe, David C. Poole, Stephanie E. Hall; data collection, Zachary J. White, Keshari H. Sudasinghe, Stephanie E. Hall; validation, Keshari H. Sudasinghe, Zachary J. White, Stephanie E. Hall; formal analysis, Zachary J. White, Stephanie E. Hall; writing—original draft preparation, Zachary J. White; writing—review and editing, Zachary J. White, Keshari H. Sudasinghe, David C. Poole, Stephanie E. Hall; funding acquisition, David C. Poole, Stephanie E. Hall. All authors have read and approved the final version of this manuscript and agree to be accountable for all aspects of the work in ensuring that questions related to the accuracy or integrity of any part of the work are appropriately investigated and resolved. All persons designated as authors qualify for authorship, and all those who qualify for authorship are listed.

## CONFLICT OF INTEREST

None declared.

## Data Availability

The data presented in this study are available on request from the corresponding author.
